# Altering the Optical Properties of GaAsSb-Capped InAs Quantum Dots by Means of InAlAs Interlayers

**DOI:** 10.1186/s11671-019-2877-2

**Published:** 2019-02-01

**Authors:** A. Salhi, S. Alshaibani, Y. Alaskar, H. Albrithen, A. Albadri, A. Alyamani, M. Missous

**Affiliations:** 1 School of Electrical and Electronic Engineering, The University of Manchester, Sackville Street, Manchester, M13 9PL UK; 2 National Center for Nanotechnology and Advanced Materials, KACST, Riyadh, 11442 Saudi Arabia; 3 Department of Physics and Astronomy, College of Sciences and Aramco Laboratory for Applied Sensors Research, KAIN, King Saud University, Riyadh, 11451 Saudi Arabia

**Keywords:** Quantum dots, Strain, InAlAs/GaAsSb, III–V semiconductors

## Abstract

In this work, we investigate the optical properties of InAs quantum dots (QDs) capped with composite In_0.15_Al_0.85_As/GaAs_0.85_Sb_0.15_ strain-reducing layers (SRLs) by means of high-resolution X-ray diffraction (HRXRD) and photoluminescence (PL) spectroscopy at 77 K. Thin In_0.15_Al_0.85_As layers with thickness *t* = 20 Å, 40 Å, and 60 Å were inserted between the QDs and a 60-Å-thick GaAs_0.85_Sb_0.15_ layer. The type II emissions observed for GaAs_0.85_Sb_0.15_-capped InAs QDs were suppressed by the insertion of the In_0.15_Al_0.85_As interlayer. Moreover, the emission wavelength was blueshifted for *t* = 20 Å and redshifted for *t* ≥ 40 Å resulting from the increased confinement potential and increased strain, respectively. The ground state and excited state energy separation is increased reaching 106 meV for *t* = 60 Å compared to 64 meV for the QDs capped with only GaAsSb SRL. In addition, the use of the In_0.15_Al_0.85_As layers narrows significantly the QD spectral linewidth from 52 to 35 meV for the samples with 40- and 60-Å-thick In_0.15_Al_0.85_As interlayers.

## Background

In the last decades, self-organized quantum dots (QDs) synthesized using the Stranski–Krastanov technique have attracted a great deal of attention. Their optical and electronic properties have been investigated intensively owing to their potential applications in optoelectronic devices [1]. The widely investigated InAs/GaAs QD system has been employed in a range of optoelectronic devices as active material. During the growth of these nanostructures, significant change in the size and the shape of the QDs occurs during the capping process. This process is quite complex and involves intermixing, segregation, or strain-enhanced diffusion [2]. The use of a pure GaAs capping layer limits the QD emission to less than 1300 nm. To alleviate this issue, strain-reducing layers made of (Ga, In)(As, Sb, N) have been used [2–7]. In particular, the ternary GaAsSb has received particular attention as its resulting band alignment can be tailored to be of type I or type II by changing the Sb content [8, 9] and by its capability in extending the emission wavelength beyond the C-band [10]. However, the difference in energy between the fundamental and excited state is limited to 60–75 meV when GaAsSb is used as a strain-reducing layer (SRL) [11]. This energy separation does not prevent carriers from escaping thermally from the QDs. For applications requiring a long carrier lifetime, the insertion of a thin barrier between the InAs QDs and GaAsSb will be beneficial, as it will increase the carrier separation between the QDs and GaAsSb quantum well (QW). As an example, GaAs interlayers have been used resulting in an enhancement of solar cell power efficiency by a factor of 23% [12]. The use of InAlAs layers may be of interest to engineering the type of radiative recombination. For type II transition, the insertion of InAlAs will increase the carrier lifetime [13] and the energy separation between the fundamental and first excited states [14–16]. Moreover, the insertion of an InAlAs layer between InAs QDs and GaAsSb is expected to decrease In segregation and suppress In and Ga atoms intermixing between the InAs QDs and the GaAsSb SRL and reduce further the QD strain [17]. InAlAs/InGaAs composite SRLs have been used to cap InAs QDs resulting in long wavelength emission and a favorable energy separation between the fundamental and excited state as high as 104 meV [16, 18].

In this paper, we report the first investigation of the effect of using an In_0.15_Al_0.85_As interlayer on the optical properties of InAs/GaAs_0.85_Sb_0.15_ QDs by means of photoluminescence (PL) spectroscopy. In particular, the emission wavelength variation, the type of optical emission, the spectral linewidth, and the energy separation between the fundamental and first excited state were studied in details.

## Methods

**Fig. 1:**
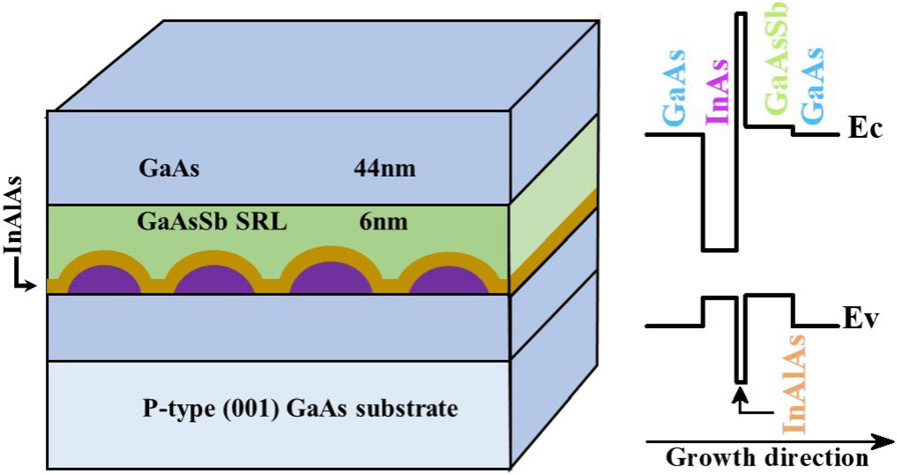
Schematic of the grown structures and corresponding energy band diagram of InAs QDs capped with a composite In_0.15_Al_0.85_As /GaAs_0.85_Sb_0.15_. The In_0.15_Al_0.85_As thickness *t* = 0 Å, 20 Å, 40 Å, and 60 Å for samples A, B, C, and D, respectively

The samples investigated in the present study were grown on epi-ready quarter 2″ p-type GaAs (001) substrates in a Veeco Gen20A Molecular Beam Epitaxy system. Valved crackers were used to generate As_2_ and Sb_2_ dimers. Following the growth of a GaAs buffer layer at 590 °C, the substrate temperature was then lowered to ~ 485 °C to grow a nominally 2.5-ML-thick InAs QDs. After a short pause under As_2_ flux, a composite In_0.15_Al_0.85_As/GaAsSb SRL was deposited immediately followed by the growth of a 5-nm-thick GaAs at the same temperature after which the growth temperature was increased to 570 °C to grow a 38-nm GaAs barrier layer. The thickness of GaAsSb was fixed at 60 Å while the thickness of In_0.15_Al_0.85_As was varied from 20 to 60 Å. The 60-Å-thick GaAsSb SRL was realized by using a As_2_/Sb_2_ flux ratio giving a Sb content of 15% as determined by X-ray diffraction measurements on a reference sample. A fixed Ga growth rate of 0.5ML/s was used for all the layers. Four samples denoted as A, B, C, and D were grown for which the In_0.15_Al_0.85_As thickness *t* was set to 0 Å, 20 Å, 40 Å, and 60 Å, respectively. Based on the procedure used by Krijn [19] and using the parameters in [20], the relative position of the conduction and valence bands have been estimated and a schematic of the grown structures with their corresponding band diagram are depicted in Fig. 1.

The crystal quality of the samples was characterized by high-resolution X-ray diffraction (HRXRD) using a Panalytical X-ray diffractometer. The optical properties of the grown samples were assessed by means of PL spectroscopy at 77 K using a PL module connected to a Vertex 80 Fourier Transform Infrared instrument (Bruker Optics GmbH) and using a thermoelectrically cooled high-gain InGaAs detector [21]. The samples were excited with a CW 532-nm solid-state laser source.

## Results and Discussion

**Fig. 2:**
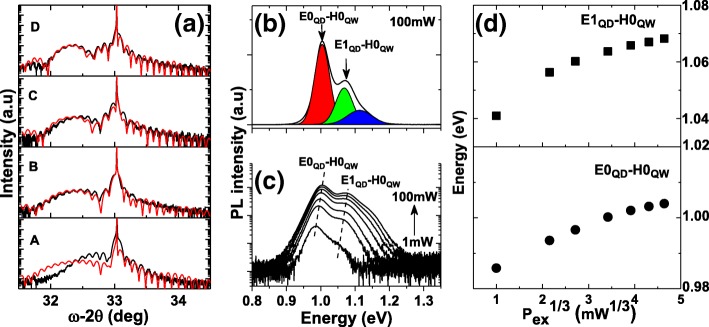
**a** High-resolution *ω*/2*θ* scans for samples A, B, C, and D. **b** PL spectrum of sample A obtained at 77 K and 100 mW excitation. **c** Power-dependent PL of sample A at 77 K. **d** The corresponding energy peak for the first two optical transitions versus P_ex_^1/3^ at 77 K

The crystal quality of the grown samples were characterized by HRXRD using rocking curve scans by recording the diffraction pattern from 004 atomic planes. Figure 2a shows the obtained diffraction patterns for samples A, B, C, and D corresponding to InAlAs thickness of 0, 20, 40, and 60 Å, respectively. Clear satellite peaks resulting from the InAs/InAlAs/GaAsSb are observed showing the good crystal quality of the grown samples. The simulated X-ray rocking curves are included in Fig. 2a alongside the experimental data. The obtained average Sb content in the reference sample A is 13% and the thickness of GaAsSb is 66 Å. These values were used in samples B, C, and D to find the In content and the thickness of the InAlAs interlayer. The simulation showed that the average In content is 13.5% and the thickness of the InAlAs interlayer is 22 Å, 44 Å, and 65 Å in samples B, C, and D, respectively, which are close to the nominal thicknesses.

**Fig. 3:**
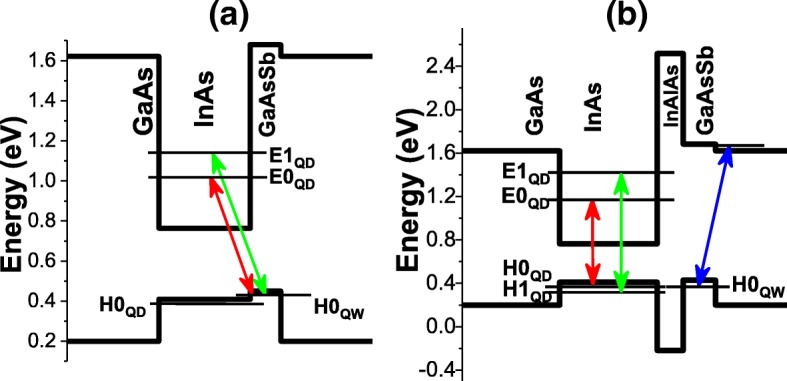
Band profiles of sample A (**a**) and samples B, C, and D (**b**) with their corresponding recombination channels

The optical properties of the reference sample A were investigated first at 77 K using the power-dependent PL technique. Figure 2b shows the PL spectrum for an excitation power of 100 mW. The PL spectrum can be fitted by three Gaussian peaks centered at 1004 meV, 1068 meV, and 1113 meV, which can be identified as the fundamental and excited optical transitions. The full width at half maximum (FWHM) of the fundamental and first excited states are 52 and 58 meV, respectively. In order to understand the origin of the observed first two optical transitions, the excitation power was varied from 1 to 100 mW and the corresponding PL spectra were acquired as depicted in Fig. 2c. For each excitation power, the energy of the first two peaks was extracted using multi-Gaussian function fitting and plotted as a function of the cube root of the excitation power as shown in Fig. 2d. The energy of the fundamental transition decreases with reducing the excitation power consistent with a type II transition indicating that the emission is a result of the recombination of electrons located in the fundamental electron state in the QD (E0_QD_) and holes located in the GaAsSb QW (H0_QW_). For InAs/GaAsSb type II band alignment, the localization of electrons and holes in the QDs and GaAsSb SRL, respectively, induces a band-bending effect resulting from the electric field, which is predominantly along the growth direction [22]. The type II transition energy is expected to increase proportionally with the third root of the excitation power as demonstrated by Jin et al. [22]. Similarly, the energy of the first excited state transition decreases with reducing the excitation power, and this transition is most likely the result of the recombination of electrons in the first electron excited state in the QD (E1_QD_) and holes within the GaAsSb QW (H0_QW_) as the density of states in the GaAsSb QW is much larger than the density of states in the QDs. The first two optical transitions are illustrated in Fig. 3a. We note also that the energy separation between the fundamental and first excited state ΔE remains nearly constant at 64 meV with decreasing excitation power, and this is evidence that the electric field resulting from the charge build up is perpendicular to the growth direction [22], i.e., the holes in the GaAsSb are localized above the QDs. A type II emission is expected in sample A as the Sb content in the GaAsSb, which is 13%, is close to the composition where a transition from type I to type II occurs [23, 24]. For the considered Sb content, a small valence band offset between the QDs and the GaAsSb QW should exist favoring the localization of holes in the GaAsSb QW and subsequently type II emissions [25, 26].

**Fig. 4:**
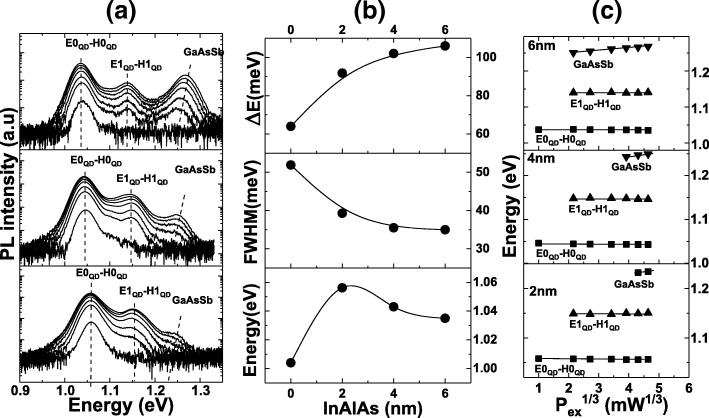
**a** Power-dependent PL of the InAs/In_0.15_Al_0.85_As/GaAsSb QDs at 77 K. **b** The corresponding peak energy, FWHM, and ΔE versus InAlAs thickness and **c** the variation of the peak energy of optical transition channels as a function of the cube root of the excitation power for samples B, C, and D

Figure 4a shows the PL emission corresponding to the samples with different In_0.15_Al_0.85_As thickness with excitation power ranging from 1 to 100 mW. Three main peaks can be identified for all the samples containing an In_0.15_Al_0.85_As interlayer. We note an alteration of the energy peak positions of the different radiative channels with respect to the reference sample A. At an excitation power of 100 mW, the energy of the fundamental transition, FWHM, and the energy separation ΔE were extracted and compared to sample A. The extracted values are reported in Fig. 4b.

The ground state transition of sample A occurs at 1004 meV with a FWHM of 52 meV and an energy separation ΔE of 64 meV. Inserting 20 Å of In_0.15_Al_0.85_As (sample B) induces a blueshift of the ground state transition by 52 meV. The blueshift is consistent with what have been observed when a composite InAlAs/InGaAs was used for QDs grown at nearly the same growth temperature [27]. The ground state transition energy blueshift of the InAs QDs in sample B results from the increased confinement potential [15]. As the barrier for electrons and holes are increased, the energy level separation of electrons and holes should increase leading to the observed emission blueshift. It is well known that capping InAs with GaAs results in a reduction of the QD height as a consequence of In segregation and In-Ga intermixing [28]. The introduction of Sb in the GaAs capping layer reduces the QD decomposition by inhibiting the strain driven In-Ga intermixing [29]. The insertion of the InAlAs interlayer is expected to suppress further the In segregation and In-Ga intermixing resulting from the inactivity of Al adatoms. In fact, Jun et al. [17] have shown by means of STEM that the use of a InAlAs/InGaAs combination layer as a capping layer strongly suppresses In segregation, and In–Ga intermixing along the growth direction during the capping process of the InAs QDs, leading to the increased height of the nanostructures and a higher In concentration in InAs QDs after capping. Considering the low growth temperature of the QDs, i.e., 485 °C, the indium segregation and interface intermixing between the QDs and InAlAs interlayer are expected to be insignificant as a result of the inactivity of Al adatoms.

The FWHM and ΔE are reduced and increased to 39 meV and 92 meV, respectively. Increasing further the thickness of In_0.15_Al_0.85_As to 40 Å and 60 Å (samples C and D, respectively) causes a redshift of the emission wavelength. This redshift is probably driven by the modification of the strain in InAs QDs as the total thickness of the composite InAlAs/GaAsSb SRL increases with increasing InAlAs thickness [30]. This may change the structural dimensions of the QDs and hence modify the energy levels of electrons and holes. It seems that for samples C and D (40 Å and 60 Å, respectively), the strain effect dominates the confinement potential effect. The lowest FWHM of 35 meV and the highest energy separation ΔE of 35 meV and 106 meV were obtained respectively for sample D. The large ΔE is caused by the use of a thicker InAlAs layer and possibly an increased QD height [31, 32]. The energy separation is comparable with that obtained when a composite InAlAs/InGaAs SRL is used (104 meV) [16, 33]. The reduction of the FWHM can be understood in terms of a reduction of intermixing between In_0.15_Al_0.85_As and QDs and hence a preservation of the QD distribution. The extracted parameters are summarized in Table 1.

**Table 1:** Extracted parameters at 77 K for samples A, B, C, and D

	A	B	C	D
InAlAs (Å)	0	20	40	60
Peak energy (meV) 77 K	1004	1056	1043	1035
FWHM (meV)	52	39	35.5	35
ΔE (meV)	64	92	102	106

The PL intensity of samples B and C was increased compared to sample A; however, a strong reduction of the PL intensity was observed for sample D, i.e., a reduction by a factor of 5 compared to sample C. The reduced PL intensity results from the reduction of carrier injection from the GaAsSb layer to the QDs. In fact, during illumination, numerous carriers are photogenerated and the insertion of the In_0.15_Al_0.85_As interlayer creates a barrier for carriers and may limit their injection in the QDs. Carriers may transfer to the QDs through a tunneling process, and the PL intensity is higher for the samples with thinner In_0.15_Al_0.85_As barriers [34]. Sample D showed the lowest PL intensity as the tunneling through the 60 Å In_0.15_Al_0.85_As is greatly reduced, and this is evidenced by the increased PL emission of the GaAsSb QW as shown in Fig. 4a. The reduction of the tunneling process makes favorable and enhances the radiative recombination of electrons and holes in the GaAsSb QW.

The main observation from the power-dependent PL at 77 K for samples B, C, and D shown in Fig. 4a is the fixed energy positions of the first two peaks with increasing excitation power as opposed to what was observed in sample A. This is a characteristic of a type I emission where both electrons and holes are localized within the QDs. The first two emission peaks result from the recombination of electrons and holes in the fundamental and first excited states in the QDs (E0_QD_-H0_QD_ and E1_QD_-H1_QD_). We believe that the third peak originates from a type II emission resulting from the recombination of electrons within GaAs and holes localized in GaAsSb QW. Indeed, the energy corresponding to this transition increases with increasing excitation power as shown in Fig. 4a and Fig. 4c characteristic of a type II transition. Moreover, we see that its intensity increases with increasing In_0.15_Al_0.85_As layer thickness. This is in agreement with the reduction of the PL intensity of the fundamental transition as a thicker In_0.15_Al_0.85_As layer reduces carriers tunneling from GaAsSb to the QDs and favors the type II emission obtained from the recombination of electrons and holes located in GaAs and GaAsSb, respectively. A schematic of the recombination channels for samples B, C, and D is depicted in Fig. 3b. The suppression of the type II emission can be understood as follows. The insertion of a 20-Å In_0.15_Al_0.85_As layer increases the carrier separation between the QDs and GaAsSb QW, and as a result, the electron and hole wavefunction overlap decreases. Moreover, the fact that the Sb content in the GaAsSb content is close to the type I-type II crossover, the In_0.15_Al_0.85_As interlayer will bring the confined level in the QW (H0_QW_) below the first quantized level in the QDs (H0_QD_) as shown in Fig. 3b and hence holes captured in the QW may tunnel through the InAlAs layer making less probable the type II emission. For a thicker In_0.15_Al_0.85_As interlayer (40 Å and 60 Å), the tunneling is further reduced but the electron and hole wavefunction overlap is substantially reduced favoring the recombination of electrons in GaAs with holes in GaAsSb [13]. The optical transition of InAs/GaAsSb QDs can be tailored to the type of application requiring either short or long lifetimes. In our study, we have considered a Sb content of 13% in GaAsSb, which is close to the type I to type II transition. The insertion of an InAlAs interlayer suppressed the type II emission and increased the energy separation between the fundamental and first excited state, which is desirable for applications requiring a short carrier lifetime. The present study can also be tailored for applications requiring a long carrier lifetime. In fact, the combination of using a higher Sb content in the GaAsSb layer and the insertion of an InAlAs interlayer is expected to maintain the type II emission for thin InAlAs interlayers while increasing significantly the carrier lifetime. At the same time, the increased energy separation between the fundamental and first excited state will reduce carrier thermal escape.

## Conclusion

InAs QDs capped with composite In_0.15_Al_0.85_As/GaAs_0.85_Sb_0.15_ SRLs with varying In_0.15_Al_0.85_As thicknesses were grown and characterized. Our analysis shows that the insertion of an In_0.15_Al_0.85_As layer suppresses the observed type II emission obtained from InAs/GaAs_0.85_Sb_0.15_ QDs. Moreover, the emission wavelength is blueshifted for *t* = 20 Å and redshifted for *t* ≥ 40 Å resulting from the increased confinement potential and increased strain, respectively. A large energy separation ΔE of 106 meV was obtained for the sample with a 60-Å-thick In_0.15_Al_0.85_As interlayer. In addition, the introduction of the In_0.15_Al_0.85_As interlayer reduces significantly the FWHM from 52 meV reaching a minimum of 35 meV.

## Abbreviations

FWHM: Full width at half maximum
HRXRD: High-resolution X-ray diffraction
PL: Photoluminescence
QDs: Quantum dots
QW: Quantum well
SRLs: Strain-reducing layers
